# IgG4‐related disease: Association with a rare gene variant expressed in cytotoxic T cells

**DOI:** 10.1002/mgg3.686

**Published:** 2019-04-16

**Authors:** John H. Newman, Aaron Shaver, Jonathan H. Sheehan, Simon Mallal, John H. Stone, Shiv Pillai, Lisa Bastarache, Derek Riebau, Hugues Allard‐Chamard, William M. Stone, Cory Perugino, Mark Pilkinton, Scott A. Smith, Wyatt J. McDonnell, John A. Capra, Jens Meiler, Joy Cogan, Kelly Xing, Vinay S. Mahajan, Hamid Mattoo, Rizwan Hamid, John A. Phillips, Undiagnosed Disease Network, David R. Adams, Aaron Aday, Mercedes E. Alejandro, Patrick Allard, Euan A. Ashley, Mahshid S. Azamian, Carlos A. Bacino, Ashok Balasubramanyam, Hayk Barseghyan, Gabriel F. Batzli, Alan H. Beggs, Babak Behnam, Hugo J. Bellen, Jonathan A. Bernstein, Anna Bican, David P. Bick, Camille L. Birch, Devon Bonner, Braden E. Boone, Bret L. Bostwick, Lauren C. Briere, Donna M. Brown, Matthew Brush, Elizabeth A. Burke, Lindsay C. Burrage, Manish J. Butte, Shan Chen, Gary D. Clark, Terra R. Coakley, Cynthia M. Cooper, Heidi Cope, William J. Craigen, Precilla D'Souza, Mariska Davids, Jean M. Davidson, Jyoti G. Dayal, Esteban C. Dell'Angelica, Shweta U. Dhar, Katrina M. Dipple, Laurel A. Donnell‐Fink, Naghmeh Dorrani, Daniel C. Dorset, Emilie D. Douine, David D. Draper, Annika M. Dries, David J. Eckstein, Lisa T. Emrick, Christine M. Eng, Gregory M. Enns, Ascia Eskin, Cecilia Esteves, Tyra Estwick, Liliana Fernandez, Carlos Ferreira, Paul G. Fisher, Brent L. Fogel, Noah D. Friedman, William A. Gahl, Emily Glanton, Rena A. Godfrey, Alica M. Goldman, David B. Goldstein, Sarah E. Gould, Jean‐Philippe F. Gourdine, Catherine A. Groden, Andrea L. Gropman, Melissa Haendel, Neil A. Hanchard, Lori H. Handley, Matthew R. Herzog, Francis High, Ingrid A. Holm, Jason Hom, Ellen M. Howerton, Yong Huang, Fariha Jamal, Yong‐hui Jiang, Jean M. Johnston, Angela L. Jones, Lefkothea Karaviti, David M. Koeller, Isaac S. Kohane, Jennefer N. Kohler, Donna M. Krasnewich, Susan Korrick, Elizabeth L. Krieg, Joel B. Krier, Jennifer E. Kyle, Seema R. Lalani, C. Christopher Lau, Jozef Lazar, Kimberly LeBlanc, Brendan H. Lee, Hane Lee, Shawn E. Levy, Richard A. Lewis, Sharyn A. Lincoln, Sandra K. Loo, Joseph Loscalzo, Richard L. Maas, Ellen F. Macnamara, Calum A. MacRae, Valerie V. Maduro, Marta M. Majcherska, May Christine V. Malicdan, Laura A. Mamounas, Teri A. Manolio, Thomas C. Markello, Ronit Marom, Martin G. Martin, Julian A. Martínez‐Agosto, Shruti Marwaha, Thomas May, Allyn McConkie‐Rosell, Colleen E. McCormack, Alexa T. McCray, Jason D. Merker, Thomas O. Metz, Matthew Might, Paolo M. Moretti, Marie Morimoto, John J. Mulvihill, David R. Murdock, Jennifer L. Murphy, Donna M. Muzny, Michele E. Nehrebecky, Stan F. Nelson, J. Scott Newberry, Sarah K. Nicholas, Donna Novacic, Jordan S. Orange, James P. Orengo, J. Carl Pallais, Christina G. S. Palme, Jeanette C. Papp, Neil H. Parker, Loren D. M. Pena, Jennifer E. Posey, John H. Postlethwait, Lorraine Potocki, Barbara N. Pusey, Chloe M. Reuter, Amy K. Robertson, Lance H. Rodan, Jill A. Rosenfeld, Jacinda B. Sampson, Susan L. Samson, Kelly Schoch, Molly C. Schroeder, Daryl A. Scott, Prashant Sharma, Vandana Shashi, Edwin K. Silverman, Janet S. Sinsheimer, Kevin S. Smith, Rebecca C. Spillmann, Joan M. Stoler, Nicholas Stong, Jennifer A. Sullivan, David A. Sweetser, Queenie K.‐G. Tan, Cynthia J. Tifft, Camilo Toro, Alyssa A. Tran, Tiina K. Urv, Zaheer M. Valivullah, Eric Vilain, Tiphanie P. Vogel, Daryl M. Waggott, Colleen E. Wahl, Nicole M. Walley, Chris A. Walsh, Melissa Walker, Jijun Wan, Michael F. Wangler, Patricia A. Ward, Katrina M. Waters, Bobbie‐Jo M. Webb‐Robertson, Monte Westerfield, Matthew T. Wheeler, Anastasia L. Wise, Lynne A. Wolfe, Elizabeth A. Worthey, Shinya Yamamoto, Yaping Yang, Amanda J. Yoon, Guoyun Yu, Diane B. Zastrow, Chunli Zhao, Allison Zheng

**Affiliations:** ^1^ Vanderbilt Center for Undiagnosed Disease Vanderbilt University Nashville Tennessee; ^2^ Department of Pathology, Microbiology and of Immunology Vanderbilt University Nashville Tennessee; ^3^ Department of Biochemistry and Center for Structural Biology Vanderbilt University Nashville Tennessee; ^4^ Department of Medicine Center for Translational Immunology and Infectious Diseases Vanderbilt University Nashville Tennessee; ^5^ Department of Medicine Massachusetts General Hospital Harvard Medical School Boston Massachusetts; ^6^ Ragon Institute of MGH MIT and Harvard Medical School Boston Massachusetts; ^7^ BioVU Vanderbilt University Medical Center Vanderbilt University Nashville Tennessee; ^8^ Department of Neurology Vanderbilt University Nashville Tennessee; ^9^ Department of Pediatrics Division of Medical Genetics Vanderbilt University Nashville Tennessee

**Keywords:** cytotoxic lymphocytes, heritable, IgG4‐RD

## Abstract

**Background:**

Family screening of a 48‐year‐old male with recently diagnosed IgG4‐related disease (IgG4‐RD) revealed unanticipated elevations in plasma IgG4 in his two healthy teenaged sons.

**Methods:**

We performed gene sequencing, immune cell studies, HLA typing, and analyses of circulating cytotoxic CD4+ T lymphocytes and plasmablasts to seek clues to pathogenesis. DNA from a separate cohort of 99 patients with known IgG4‐RD was also sequenced for the presence of genetic variants in a specific gene, FGFBP2.

**Results:**

The three share a previously unreported heterozygous single base deletion in fibroblast growth factor binding protein type 2 (FGFBP2), which causes a frameshift in the coding sequence. The FGFBP2 protein is secreted by cytotoxic T‐lymphocytes and binds fibroblast growth factor. The variant sequence in the FGFBP2 protein is predicted to form a disordered random coil rather than a helical‐turn‐helix structure, unable to adopt a stable conformation. The proband and the two sons had 5–10‐fold higher numbers of circulating cytotoxic CD4 + T cells and plasmablasts compared to matched controls. The three members also share a homozygous missense common variant in FGFBP2 found in heterozygous form in ~40% of the population. This common variant was found in 73% of an independent, well characterized IgG4‐RD cohort, showing enrichment in idiopathic IgG4‐RD.

**Conclusions:**

The presence of a shared deleterious variant and homozygous common variant in FGFBP2 in the proband and sons strongly implicates this cytotoxic T cell product in the pathophysiology of IgG4‐RD. The high prevalence of a common FGFBP2 variant in sporadic IgG4‐RD supports the likelihood of participation in disease.

## 1 INTRODUCTION

IgG4‐RD is an emerging multi‐organ inflammatory illness for which the pathogenesis is poorly defined (Perugino et al., 2017; Stone, Zen, & Deshpande, 2012). Recent work on mechanistic aspects of this disease has identified the importance of an oligoclonally expanded CD4+ cytotoxic T lymphocyte population. CD4+ T cells with a cytolytic phenotype (CD4 + CTLs) are clonally expanded in patients with IgG4‐RD (Mattoo et al., 2016) and are likely to be essential to the pathophysiology of the disease. These cells secrete pro‐fibrotic cytokines including IL‐1beta, transforming growth factor‐beta, and interferon‐gamma, and are capable of perforin‐ and granzyme B‐mediated cytolysis. They accumulate in tissue lesions, representing the dominant T cell population in these affected tissues (Maehara et al., 2017; Mattoo et al., 2016). Patients with IgG4‐RD also have elevations of circulating plasmablasts (Mattoo et al., 2014; Wallace et al., 2015). Because of the profound clinical responses and decline in CD4 + CTLs observed following B‐cell depleting therapy, it is considered likely that activated B cells in tissues drive the activation of cytotoxic CD4 + T cells in affected tissues (Maehara et al., 2017; Mattoo et al., 2016; Perugino et al., 2017).

We report a family study of a father with IgG4‐RD and two sons who also have elevated plasma IgG4 concentrations. The three share a newly described gene variant in fibroblast growth factor binding protein 2 (*FGFBP2*), OMIM: 607713, and markedly elevated blood plasmablasts and CD4 + cytotoxic T cells. Heritable IgG4‐RD has been inferred in prior reports, but no information exists on genetic features that may predispose to disease (Comings, Skubi, Van Eyes, & Motulsky, 1967; Matzumura, Rubin, & Meysami, 2017).

## 2 CLINICAL REPORT

The proband was healthy until age 37 when he noted gradual swelling under his mandible and around the eyes. Evaluation prior to referral to the UDN included biopsies from submandibular salivary and lacrymal glands that showed follicular hyperplasia without evidence of lymphoma. Other medical history included chronic sinusitis, mild intermittent asthma, type II diabetes mellitus, hypothyroidism, and transient ischemic attacks in a middle cerebral artery distribution.

Prior evaluation of the transient ischemic attacks included cerebral angiography that showed a narrow left middle cerebral artery (MCA) stenosis. This was dilated by balloon angioplasty but subsequently recurred. Extensive evaluation for immune vascular disease was negative as was a survey of known causes of hypercoagulability. The patient did not have hypercholesterolemia or systemic hypertension and had no family history of cerebral vascular disease. A second MCA stenosis occurred and the patient had watershed ischemic zones in the cerebral hemisphere noted on magnetic resonance imaging (MRI). The cerebral circulation developed collaterals and a moyamoya appearance was identified on angiography (Scott & Smith, 2009). He ultimately underwent extra‐cranial MCA bypass from a temporal artery branch and has remained stroke‐free thereafter.

The patient was referred through the NIH Undiagnosed Disease Network to the Vanderbilt Center for Undiagnosed Disease (VCUD) for further evaluation. Family history was negative for any unusual diseases or of familial illness. The proband's father had resected prostate cancer and his mother had died of lymphoma. The proband had no unusual hobbies or occupational exposures and did not smoke. His childhood and adolescence had been without unusual diseases. The brother and wife had none of the proband's disease features. The proband's two children were healthy teenage boys who had normal school advancement and participation in sports. Plasma IgG4 levels were obtained on all family members and were normal except for the two sons having elevated levels at 125 mg/dl (nl < 89) and 184 mg/dl (nl < 143 for age).

### 2.1 Diagnosis of IgG4‐RD

Review of the proband's prior submandibular biopsy tissue showed a dense lymphoplasmacytic infiltrate, storiform fibrosis, and obliterative phlebitis. Immunostains of the tissue revealed a predominant IgG4‐positive plasma cell infiltrate (>100 per high power field), with an IgG4:total IgG ratio of > 40%, thereby satisfying the histopathologic criteria for a diagnosis of IgG4‐RD (Carruthers et al., 2015). The patient's plasma IgG4 level was 260 mg/dl (nl < 89). The patient was treated with rituximab with subsequent regression of the orbital and submandibular swelling.

The intracranial vascular stenosis in the proband was unusual in severity, persistence, and lack of identifiable risk factors. Although IgG4‐RD can be associated with large‐vessel vasculitis, intracranial vascular disease has not been reported (Abdelrazek, Venna, & Stone, 2018). Because Moyamoya disease is associated with variants in RNF213 in Japanese and Korean patients we sought to ascertain if the proband also had such variants (Kamada et al., 2011; Scott & Smith, 2009). The patient has no known Asian ancestry.

### 2.2 Candidate variants from WGS

Ethical considerations: this study was approved by the Institutional Review Board of the NIH for the UDN and the proband and family gave informed consent for studies, research, and report. Whole genome sequencing (WGS) was performed at the UDN sequencing core on the proband, his wife, father, brother, and two teenage sons. No clinically actionable genes were found. WGS revealed two candidate variants that were shared by the proband and his two sons. These variants were also shared with the proband's normal father, but not the brother or wife.

The first is a previously unreported frameshift variant, NM_031950.3:c.602delA p.Lys201ArgfsTer26, in fibroblast growth factor binding protein type 2 (*FGFBP2*), chromosome 4, OMIM:614768. *FGFBP2* is expressed in cytotoxic lymphocytes whose function is unclear. Given the fact that CD+CTLS have been implicated by us in this disease, we speculate that an alteration in FGFBP2 protein might enhance cytotoxic CD4 + T cell function. We have no information on FGFBP2 protein and IgG4 class switch.

A second variant was found in *FGFBP2* c.268C>Tp.Pro90Ser (rs758329) OMIM: 607713 that has an allele population frequency of 0.40 in the ExAC database. This common missense variant was homozygous in the proband, two sons and father, and heterozygous in the proband's wife.

We searched for *FGFBP2* variants in a large repository of 99 known IgG4‐RD patients at the Massachusetts General Hospital (MGH). Complete sequencing of the *FGFBP2* gene was done in 51 subjects with IgG4‐RD and in a validation cohort of 48 patients. Although the rare missense variant found in our family was not detected in any of the MGH subjects, the common *FGFBP2* variant, rs758329 was enriched in the MGH test IgG4‐RD cohort (76.2%, compared to 40.3% for the population at large). The validation cohort of 48 IgG4‐RD subjects also revealed enrichment of rs758329 (70.8%). All other SNPs remained at close to population frequencies for large numbers of non‐Finnish European populations. Thus, the common variant was not only enriched in nonfamilial IgG4‐RD but also homozygous in our affected patients.

A variant in a gene associated with Moyamoya disease was found in the heterozygous state in the proband, his father and two sons but not in the brother or wife. Variants in this gene, RNF 213, chr 17 are associated with disease in about 15% of the Moyamoya cases in Japan and Korea (Kamada et al., 2011). This missense variant, OMIM:613768, NM_001256071.1;C13250B>A;plArg4417His, rs150148627, is found in 6 in 10,000 in the ExAC data base (Kamada et al., 2011), but has not been associated with Moyamoya in Asians or in persons of European descent. It has no known association with the FGFBP2 protein. Because a vascular biopsy was not indicated during the MCA bypass, we do not have information on the pathology of the patient's cerebral vascular disease and whether it might have features of IgG4‐RD. There is no reported association of RNF 213 variants and IgG4‐RD.

### 2.3 Effect of the frameshift mutation on FGFBP2 protein

**Figure 1:**
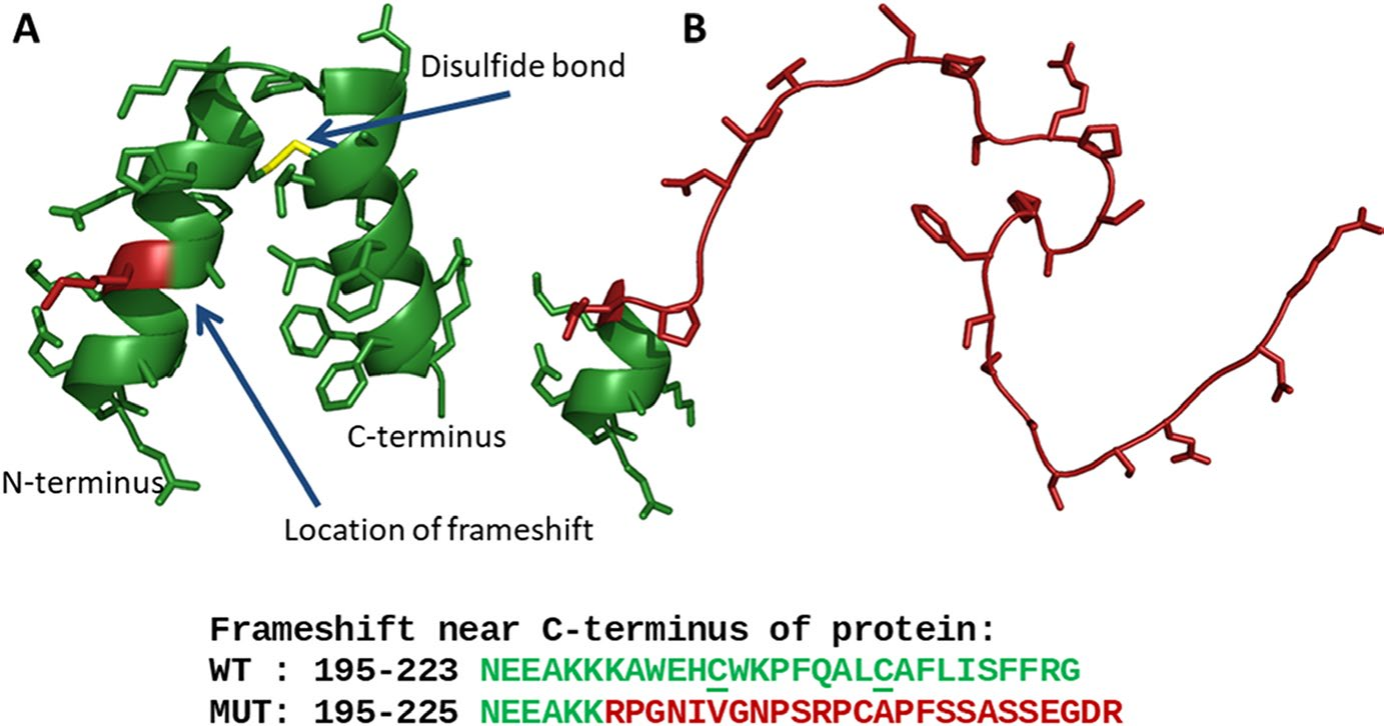
A structural model for FGFBP2The model for FGFBP2 suggests a mechanism for the effect of the frameshift variant. (a) The C‐terminal domain is predicted to form two alpha helices held at an angle by a conserved disulfide bond. Protein backbone is shown as green ribbons; sidechains are shown as sticks; position of frameshift is highlighted in red. (b) The frameshifted sequence is predicted to form a disordered random coil

In order to understand the mechanism by which the rare variant would alter the function of FGFBP2, we applied sequence analysis and structural modeling to the wild‐type (WT) and variant (MUT) protein sequences, Figure 1. The frameshift changes the C‐terminal sequence of the protein from NEEAKKKAWEHCWKPFQALCAFLISFFRG (residues 195‐223 in WT) to AKKRPGNIVGNPSRPCAPFSSASSEGDR (residues 195‐225 in MUT). The model does not cover the entire protein, because the N‐terminal section is not affected by the frameshift. The WT domain is predicted to form a pair of 14 residue alpha helices held at an angle by a disulfide bond linking C‐206 to C‐214. Three functional consequences are predicted from this alteration in the variant. First, the original function of the C‐terminal domain (e.g., protein‐binding) will be lost because the functional structure is no longer present to carry it out. Second, the stretch of 25 disordered amino acids has an increased probability of aggregation or degradation. And, third, the remaining unpaired cysteine residue has the potential to make adventitious bonds before or after secretion from the cell. Because the variant is not predicted to undergo nonsense‐mediated decay, the presence of the remaining intact N‐terminal domain attached to the nonfunction C‐terminal alteration might be sufficient to act in a dominant negative manner. These predictions remain to be tested experimentally.

### 2.4 Effect of the missense variant on protein in RNF 12

Structural modeling by similar techniques as above was used to determine the effects of the missense variant on the RNF protein. The protein is a ubiquitin‐protein ligase that may play a role in angiogenesis, involved in Wnt signaling. The mutation in our family falls within a structured region that alternates helix and loop. A nearby mutation is known to increase the risk of Moyamoya disease (Kamada et al., 2011). Modeling of this region suggests that these two residues do not face each other. Thus, it is not possible to infer that the variant in our family is likely to alter protein function. A search in the Vanderbilt BioVU and synthetic derivative found 28 subjects with rs150148627 variant, but none had intracranial vascular disease pointing to a phenotype similar to our patient. Any relationship of this RNF variant to the moyamoya and the IgG4‐RD in our patient is speculative.

Secondary structure predictions of WT and MUT sequences were performed using PSIPRED v3.3 at bioinf.cs.ucl.ac.uk/psipred/(Jones, 1999). Disorder predictions were performed using DISOPRED3 at the same site (Buchan, Minneci, Nugent, Bryson, & Jones, 2013). Tertiary structure predictions were performed using the PEP‐FOLD3 server at bioserv.rpbs.univ‐paris‐diderot.fr/services/PEP‐FOLD3/(Lamiable et al., 2016). Structural figures of the WT and MUT were prepared using PyMOL v1.8.2.

### 2.5 Immune studies

An HLA family study did not reveal a shared haplotype among the father (A*68:01, B*35:03, C*04:01, DRB1*07:01 and A*34:02, B*44:02, C*05:01, DRB1*13:01) and two sons (son 1, A*34:02, B*44:02, C*05:01, DRB1*13:01; A*02:01, B*57:01, C*06:02, DRB1*11:01; and son 2 A*68:01, B*35:03, C*04:01, DRB1*07:01; A*03:01, C*07:02, B*07:02, DRB1*15:01).

### 2.6 TCR sequencing of involved lymph node from Proband

A formalin‐fixed, paraffin‐embedded lymph node section was processed for T cell receptor sequencing by Adaptive Biotechnologies. Fifteen thousand eight hundred and eight‐five total T cells had a productive TCR; of those T cells, 13,313 had a unique CDR3 region. Analysis of this lymph node T cell receptor repertoire revealed a highly diverse T cell repertoire, as would be expected of the lymph node. However, repertoire motif and permutation analysis using the GLIPH algorithm (Glanville et al., 2017) revealed that the most dominant clone (CASSFRRGSVQLNEQFF, using TRBV‐11*02 and TRBJ‐02*01, contributing 202 T cells for a total of 1.2% of the sample) was clonally expanded at 15‐fold above chance alone (*p* < 0.001), suggesting an antigen‐driven expansion. This dominant TCR was four times more frequent than the next most frequent CDR3. The overwhelming majority of TCRs was unique minority species and contributed less than T cells each. A similar Next Gen sequencing approach has revealed that patients with IgG4‐related disease have clonal expansions in the blood of CD4 + CTLs (Carruthers et al., 2015).

### 2.7 Flow cytometry for circulating plasmablasts and CD4 + CTLs

Briefly, PBMCs were thawed and Fc blocked on ice for 15 minutes using Human TruStain FcX™ (Biolegend). Fluorescence labeling was then performed for 30 minutes on ice using monoclonal antibodies from Biolegend unless otherwise specified. Plasmablast identification was undertaken using the following antibodies: FITC anti‐human CD3 (clone UCHT1), APC/Cy7 anti‐human CD19 (clone SJ25C1), APC anti‐human CD79b (Igβ) (clone CB3‐1), PerCP/Cy5.5 anti‐human CD20 (clone 2H7), Brilliant Violet 510™ anti‐human CD27 (clone M‐T271), and PE/Cy7 anti‐human CD38 (clone HIT2).

Antigen‐experienced/effector CD4 + T cells and CD4 + CTLs were identified using the following antibodies: Alexa Fluor® 488 anti‐human CD4 (clone OKT4), Brilliant Violet 510™ anti‐human CD27 (clone M‐T271), APC/Cy7 anti‐human CD45RO (clone UCHL1), and PE anti‐human CD319 (SLAMF7) (clone 162.1). All cells were counter stained using blue viability dye to exclude apoptotic cells (Thermofisher) and analyzed on a BD LSR II cytometer (BD Biosciences). Subsequent analyses were carried out using FlowJo V10.3.

**Figure 2:**
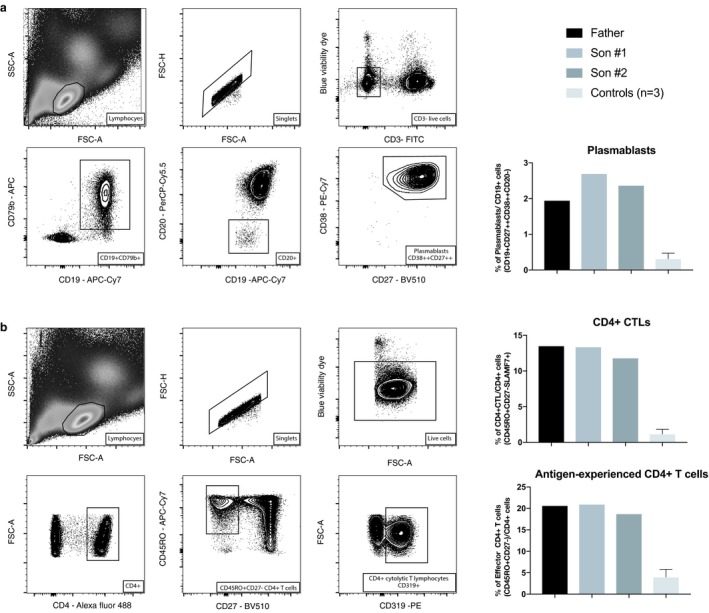
Subjects with the FGFBP2 c.268C>T p.Pro90Ser variant have increased numbers of circulating plasmablasts, effector CD4 + T cells, and CD4 + CTLs. Gating strategy for enumeration of circulating plasmablasts (a) and CD45RO+ CD27‐ effector CD4 + T cells and CD4 + CTLs (b). The three subjects (father and two sons) presented with evidence for lymphocyte activation in both B and T cell compartments. They had relatively high proportions of circulating plasmablasts (a) as well as of antigen‐experienced effector CD4 + T cells and CD4 + CTLs (b) when compared with three healthy controls

Flow cytometric enumeration of activated B and T cells was undertaken as previously described (Mattoo et al., 2014, 2016). The proband and his two sons exhibited marked elevations of circulating plasmablasts and CD4 + CTLs, comparable to those seen in patients with active IgG4‐related disease (Wallace et al., 2015) (Figure 2). What is unusual about the findings is that the two children have no disease symptoms but have elevated plasmablasts and CD4 + CTLS, suggesting that they may have preclinical disease. We have not done RNA seq but have plans to do so in this family with our expanded cohort of IgG4‐RD patients. One very tight definition of CD+ effectors is to look at CD45RO+CD62L‐CD27‐cells. This is the definition we used and previously published (Mattoo et al., 2016).

### 2.8 Frequency of IgG4 producing single B cells in family members

Minimum IgG4‐producing B frequencies of cells were calculated using PBMCs. Minimum IgG4 frequencies were defined as the number of IgG4 containing positive culture wells per 1 million PMBCs cultured. This is a minimum frequency and likely underestimates the true frequency as it does not account for the occurrence of more than one IgG4 expressing cell being present in a positive well. IgG4 was detected in culture supernatant by ELISA using the commercially available murine monoclonal antibody, HP6024. Our B cell culturing methods do not use flow cytometry or Miltenyi column B cell separation. No IgG4‐producing B cells could be cultured from the proband, who had previously been treated with rituximab. His two asymptomatic sons had minimum IgG4‐producing B cell frequencies at least fivefold higher (40, 43 per million PBMCs) than the unaffected brother of the proband (8 per million PBMCs).

## 3 DISCUSSION

CD4 + T cells with a cytolytic phenotype (CD4 + CTLs) that secrete pro‐fibrotic cytokines including IL‐1beta, TGF‐beta, and interferon‐gamma are viewed as essential to the pathophysiology of IgG4‐RD (Maehara et al., 2017; Mattoo et al., 2016). These cells, which are capable of mediating perforin and granzyme B‐mediated cytolysis, are clonally expanded in patients with IgG4‐RD (Mattoo et al., 2016)). Moreover, they accumulate in tissue lesions and represent the dominant T cell population in these tissues (Maehara et al., 2017; Mattoo et al., 2016). Patients with IgG4‐RD also have elevations of circulating plasmablasts (Wallace et al., 2015) and it is hypothesized that activated B cells and plasmablasts in tissues promote the reactivation of cytotoxic CD4 + T cells in affected tissues. The potential role of activated CD8 + T cells in the pathogenesis of this disease remains to be explored. The mechanism of the elevation of IgG4 in the two sons is not known. We have published recently that IL‐4 and BATF positive TFH cells reside in lymph nodes and lymphoid organs of patient with IgG4‐RD. The numbers show a tight correlation with the IgG4 class switch. What triggers the development of this disease‐related subset of TFH cells is not clear but we speculate that this is the cause of the increased IgG4 in the proband and sons.

In summary, we have analyzed the family of a patient with IgG4‐RD. His teenage sons, though healthy, have abnormal IgG4 levels and high levels of circulating IgG4 plasmablasts and CD4 + CTLs. The proband and sons share a rare missense variant in a protein that binds fibroblast growth factor and is predicted to reduce function. This variant was not found in a cohort of 99 cases of idiopathic IgG4‐RD, but another common FGFBP2 variant was found to be highly enriched in these known cases and was found in homozygous form in the affected members of our family. The possibility of familial IgG4‐RD seems strong, although the likelihood that the sons will develop IgG4‐RD is unknown. Further work on the functional properties and the possible contribution of the abnormal FGFBP2 protein to IgG4‐RD is needed. This is an unusual case of familial IgG4‐RD with possible involvement of a dysfunctional T cell protein.

## CONFLICT OF INTEREST

None declared.

## APPENDIX 1

### Members of the Undiagnosed Diseases Network

David R. Adams, Aaron Aday, Mercedes E. Alejandro, Patrick Allard, Euan A. Ashley, Mahshid S. Azamian, Carlos A. Bacino, Ashok Balasubramanyam, Hayk Barseghyan, Gabriel F. Batzli, Alan H. Beggs, Babak Behnam, Hugo J. Bellen, Jonathan A. Bernstein, Anna Bican, David P. Bick, Camille L. Birch, Devon Bonner, Braden E. Boone, Bret L. Bostwick, Lauren C. Briere, Donna M. Brown, Matthew Brush, Elizabeth A. Burke, Lindsay C. Burrage, Manish J. Butte, Shan Chen, Gary D. Clark, Terra R. Coakley, Cynthia M. Cooper, Heidi Cope, William J. Craigen, Precilla D'Souza, Mariska Davids, Jean M. Davidson, Jyoti G. Dayal, Esteban C. Dell'Angelica, Shweta U. Dhar, Katrina M. Dipple, Laurel A. Donnell‐Fink, Naghmeh Dorrani, Daniel C. Dorset, Emilie D. Douine, David D. Draper, Annika M. Dries, David J. Eckstein, Lisa T. Emrick, Christine M. Eng, Gregory M. Enns, Ascia Eskin, Cecilia Esteves, Tyra Estwick, Liliana Fernandez, Carlos Ferreira, Paul G. Fisher, Brent L. Fogel, Noah D. Friedman, William A. Gahl, Emily Glanton, Rena A. Godfrey, Alica M. Goldman, David B. Goldstein, Sarah E. Gould, Jean‐Philippe F. Gourdine, Catherine A. Groden, Andrea L. Gropman, Melissa Haendel, Neil A. Hanchard, Lori H. Handley, Matthew R. Herzog, Francis High, Ingrid A. Holm, Jason Hom, Ellen M. Howerton, Yong Huang, Fariha Jamal, Yong‐hui Jiang, Jean M. Johnston, Angela L. Jones, Lefkothea Karaviti, David M. Koeller, Isaac S. Kohane, Jennefer N. Kohler, Donna M. Krasnewich, Susan Korrick, Elizabeth L. Krieg, Joel B. Krier, Jennifer E. Kyle, Seema R. Lalani, C. Christopher Lau, Jozef Lazar, Kimberly LeBlanc, Brendan H. Lee, Hane Lee, Shawn E. Levy, Richard A. Lewis, Sharyn A. Lincoln, Sandra K. Loo, Joseph Loscalzo, Richard L. Maas, Ellen F. Macnamara, Calum A. MacRae, Valerie V. Maduro, Marta M. Majcherska, May Christine V. Malicdan, Laura A. Mamounas, Teri A. Manolio, Thomas C. Markello, Ronit Marom, Martin G. Martin, Julian A. Martínez‐Agosto, Shruti Marwaha, Thomas May, Allyn McConkie‐Rosell, Colleen E. McCormack, Alexa T. McCray, Jason D. Merker, Thomas O. Metz, Matthew Might, Paolo M. Moretti, Marie Morimoto, John J. Mulvihill, David R. Murdock, Jennifer L. Murphy, Donna M. Muzny, Michele E. Nehrebecky, Stan F. Nelson, J. Scott Newberry, Sarah K. Nicholas, Donna Novacic, Jordan S. Orange, James P. Orengo, J. Carl Pallais, Christina G. S. Palme, Jeanette C. Papp, Neil H. Parker, Loren D. M. Pena, Jennifer E. Posey, John H. Postlethwait, Lorraine Potocki, Barbara N. Pusey, Chloe M. Reuter, Amy K. Robertson, Lance H. Rodan, Jill A. Rosenfeld, Jacinda B. Sampson, Susan L. Samson, Kelly Schoch, Molly C. Schroeder, Daryl A. Scott, Prashant Sharma, Vandana Shashi, Edwin K. Silverman, Janet S. Sinsheimer, Kevin S. Smith, Rebecca C. Spillmann, Joan M. Stoler, Nicholas Stong, Jennifer A. Sullivan, David A. Sweetser, Queenie K.‐G. Tan, Cynthia J. Tifft, Camilo Toro, Alyssa A. Tran, Tiina K. Urv, Zaheer M. Valivullah, Eric Vilain, Tiphanie P. Vogel, Daryl M. Waggott, Colleen E. Wahl, Nicole M. Walley, Chris A. Walsh, Melissa Walker, Jijun Wan, Michael F. Wangler, Patricia A. Ward, Katrina M. Waters, Bobbie‐Jo M. Webb‐Robertson, Monte Westerfield, Matthew T. Wheeler, Anastasia L. Wise, Lynne A. Wolfe, Elizabeth A. Worthey, Shinya Yamamoto, Yaping Yang, Amanda J. Yoon, Guoyun Yu, Diane B. Zastrow, Chunli Zhao, Allison Zheng.
